# OMRT-8. Precision targeting of cellular pathways with complementary diagnostics

**DOI:** 10.1093/noajnl/vdab070.033

**Published:** 2021-07-05

**Authors:** Robert Siddaway, Liana Nobre, Scott Milos, Monique Johnson, Scott Ryall, Javal Sheth, Michelle Ku, Uri Tabori, Cynthia Hawkins

**Affiliations:** Hospital for Sick Children, Toronto, ON, Canada

## Abstract

Precision medicine tailors treatment for each patient by identifying the molecular drivers of their disease. This can allow more effective tumour targeting, avoid harmful standard chemotherapeutic side-effects, and offer savings to the healthcare system through not treating patients who are unlikely to respond to a specific agent. Treatment regimes are usually designed by identifying DNA-level alterations and selecting drugs tailored to that mutation. However, cancer is not a one-pathway disease and not all patients with particular mutations will respond to treatment, while patients without canonical pathway-activating mutations are excluded from potentially life-saving treatment. To address this, we have developed a NanoString assay combining proteomic and transcriptomic profiles of 4 key actionable, cancer-related pathways (MAPK, PI3K, NFκB and JAK/STAT). We used RNA-Seq data from gold standard cell lines with defined pathway changes to identify minimal gene sets indicative of pathway activation, and integrated them with phospho protein measurements to generate a pathway activation score. The combined panel was run on isogenic cell lines as well as glioma samples with both known and unknown driving alterations. We found pathway activation to be more variable than expected based on DNA alterations alone, implying that consideration of proteomic and/or transcriptomic-level information is important for future therapeutic decision-making.

